# CANcer-specific Evaluation System (CANES): a high-accuracy platform, for preclinical single/multi-biomarker discovery

**DOI:** 10.18632/oncotarget.19270

**Published:** 2017-07-15

**Authors:** Min-Seok Kwon, Seungyoon Nam, Sungyoung Lee, Young Zoo Ahn, Hae Ryung Chang, Yon Hui Kim, Taesung Park

**Affiliations:** ^1^ Interdisciplinary Program in Bioinformatics, Seoul National University, Seoul 08826, Korea; ^2^ Gachon Institute of Genome Medicine and Science, Gachon University Gil Medical Center, Incheon 21565, Korea; ^3^ Department of Life Sciences, Gachon University, Seongnam-si 13120, Korea; ^4^ College of Medicine, Gachon University, Incheon 21565, Korea; ^5^ System Cancer Science, Graduate School of Cancer Science and Policy, National Cancer Center of Korea, Goyang-si 10408, Korea; ^6^ Research Institute of Women’s Health, Sookmyung Women’s University, Seoul, 04310, Korea; ^7^ Corestem Inc., Seongnam-si 13486, Korea; ^8^ Department of Statistics, Seoul National University, Seoul 08826, Korea

**Keywords:** biomarker performance, claudin, gastric cancer, transcriptome, data mining

## Abstract

The recent creation of enormous, cancer-related “Big Data” public depositories represents a powerful means for understanding tumorigenesis. However, a consistently accurate system for clinically evaluating single/multi-biomarkers remains lacking, and it has been asserted that oft-failed clinical advancement of biomarkers occurs within the very early stages of biomarker assessment. To address these challenges, we developed a clinically testable, web-based tool, CANcer-specific single/multi-biomarker Evaluation System (CANES), to evaluate biomarker effectiveness, across 2,134 whole transcriptome datasets, from 94,147 biological samples (from 18 tumor types). For user-provided single/multi-biomarkers, CANES evaluates the performance of single/multi-biomarker candidates, based on four classification methods, support vector machine, random forest, neural networks, and classification and regression trees. In addition, CANES offers several advantages over earlier analysis tools, including: 1) survival analysis; 2) evaluation of mature miRNAs as markers for user-defined diagnostic or prognostic purposes; and 3) provision of a “pan-cancer” summary view, based on each single marker. We believe that such “landscape” evaluation of single/multi-biomarkers, for diagnostic therapeutic/prognostic decision-making, will be highly valuable for the discovery and “repurposing” of existing biomarkers (and their specific targeted therapies), leading to improved patient therapeutic stratification, a key component of targeted therapy success for the avoidance of therapy resistance.

## INTRODUCTION

Traditionally, biomarker studies begin from a handful number of candidate genes or proteins, based on experimental and computational assessment. Also, for given candidates, validation techniques, and their supporting evidence, have been compromised, due to a lack of technical advances and publicly available clinical data. Now that various technologies, including next-generation sequencing, are mature, it is possibly to rapidly analyze “Big Data” (e.g., whole tumor transcriptomes and genomes) for association with clinical information. However, while these high-technology approaches should empower clinical researchers to identify clinical, translational, and accessible biomarkers, few approaches for this purpose have been successful [1]. To overcome the challenges of biomarker-driven cancer therapy, various standards and guidelines have been made to increase the rigor of the development process [2]. For screening purposes, diagnostic biomarkers would require a generally agreed upon requirement of high specificity and sensitivity, to allow general population screening for even the most common cancers [3]. For example, it is estimated that for a relatively rare disease such as ovarian cancer (1.3% lifetime risk), effective (i.e., resulting in reduced mortality) screening, for an asymptomatic population of 2500 women, would require a sensitivity ≥ 75% and a specificity ≥ 99.6%, to achieve a positive predictive value of 10% for the detection of individuals with stage 1 disease (at which the disease is > 90% curable) [4]. To achieve such predictive accuracy, it has been asserted that combinations of biomarkers (“biomarker panels”) may allow obtainment of such stringent criteria [5, 6].

There are more than 200 types of cancer from over 60 different organs in the body [7]. Some cancers of different organs have many shared features, such as therapeutic response, while conversely, some cancer subtypes from the same organ are quite distinct [8]. These phenotypic features of cancer types depend on the expression patterns of single or multiple genes [9, 10]. For example, since the oncogene *ERBB2* (*HER2*) is amplified in subgroups of glioblastoma and, stomach, uterine, bladder, and lung cancers, responsiveness to HER2-targeted therapy may or may not be analogous to that of *HER2*-amplified breast cancer [9, 10]. Similarly, erlotinib, an effective inhibitor of the actively mutated epidermal growth factor receptor (EGFR), originally approved for the treatment of advanced pancreatic cancer, has now shown efficacy for non-small cell lung and various other cancers [11]. Here, to more rapidly make such preliminary determinations, we designed and developed a comprehensive web-based assessment tool, “CANcer-specific Evaluation System” (CANES), for exhaustive biomarker evaluation that: (i) employs repositories across 2,134 whole transcriptome datasets, from 94,147 biological samples (cell lines and normal and cancerous tissues), representing 18 tumor types; (ii) performs the initial steps of evaluating single and/or multi-genes as biomarker candidates; and (iii) uses various classification methods to support diagnostic or prognostic assessment of genes, as well as miRNAs, as biomarkers, yielding a “pan-cancer” summary view of the evaluation of each individual biomarker. Finally, one of the outstanding features of CANES is that it allows direct comparison between the diagnostic or prognostic performance of single vs. multi-biomarker sets. Multi-biomarker sets often tend to show good performance, by chance, when the number of biomarkers is large and sample size is small, resulting in artifactual results. CANES addresses this problem by providing standardized evaluation measures and empirical p-values, allowing direct comparison of the diagnostic/prognostic performance of multi-biomarker sets, having different numbers of biomarkers.

In summary, CANES represents a powerful tool for “landscape” evaluation across 18 cancer-types for single/multi-biomarkers, in association with diagnostic therapeutic decision making and prognostic use by preclinical researchers, producing high-quality results that can be further translated toward clinical “precision medicine.”

## RESULTS

### Demonstration of CANES performance in predicting single vs. multi-biomarker evaluation

**Figure 1:**
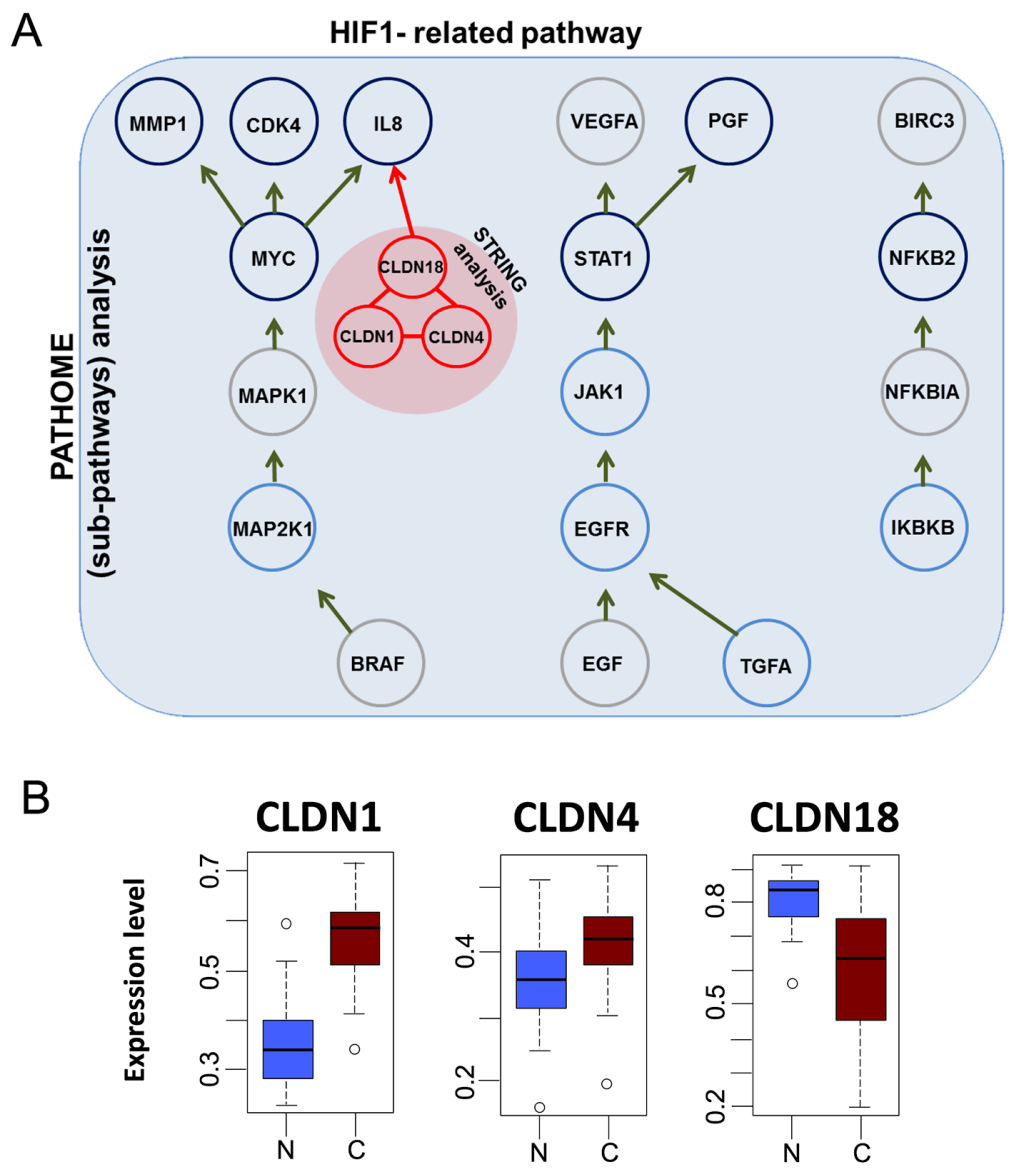
The figure here depicts HIF-1 pathway and gene expression levels of *CLDN1*, *CLDN4*, and *CLDN18* in gastric cancer **(A)** HIF-1-related pathway (previously identified by our established algorithm (12) showing additional interactions with a triad consisting of *CLDN1*, *CLDN4*, and *CLDN18*, as identified by the STRING database (indicated in the red-filled circle). **(B)** Gene expression levels of *CLDN1*, *CLDN4*, and *CLDN18*, in gastric cancer (“C”) vs. normal (“N”) samples in a gastric cancer dataset (GSE13911). *CLDN1* and *CLDN4* were upregulated in tumors, while CLDN18 was downregulated.

In our previous study, we identified several pathways involved in gastric cancer progression using our systems biology approach, PATHOME [12]. We also showed the significance of regulation of HNF4α, as well as reduced HIF1α, in early gastric cancer (GC) [12, 13], as detected only by our PATHOME algorithm (Figure 1A). We further found the HIF1-related pathway to associate with three claudin protein family members (claudins-1, -4, and -18), by a protein-protein interaction tool, STRING (Search Tool for the Retrieval of Interacting Genes/Proteins, version 9.1) [14] (Figure 1A). Of the three claudins we identified to interact with an HIF-1 network, *CLDN1* and *CLDN4* were previously reported as upregulated in gastric cancer progression, while *CLDN18* was downregulated [15–20]. Our CANES results were consistent with those previously shown gene expression patterns in another GC dataset (GSE13911) (Figure 1B). Table 1 shows that for biomarker use of the three *CLDN* genes for gastric cancer, *CLDN18* had the highest balanced accuracy (BA), followed by *CLDN1* and then *CLDN4*.

**Table 1:** Prediction measures of three single markers (*CLDN1*, *CLDN4*, and *CLDN18*) and a multi-marker (*CLDN1*/*4*/*18*) for gastric cancer (cancer tissue vs. normal tissue)

Average evaluation measure	Single marker			Multi-marker
	*CLDN1*	*CLDN4*	*CLDN18*	(*CLDN1*, *4*, *18*)
**Area under curve (AUC)**	0.756	0.647	0.792	0.850
**Balanced accuracy (BA)**	0.776	0.659	0.801	0.851
**Accuracy (AC)**	0.892	0.784	0.905	0.936
**Sensitivity (SN)**	0.817	0.705	0.880	0.849
**Specificity (SP)**	0.696	0.590	0.705	0.851
**Positive predictive value (PPV)**	0.821	0.693	0.778	0.859
**Negative predictive value (NPV)**	0.618	0.548	0.855	0.851
**False positive rate (FPR)**	0.304	0.409	0.295	0.149
**False discovery rate (FDR)**	0.178	0.306	0.221	0.150
**F1 score (F1)**	0.789	0.690	0.822	0.849

These performance measures are for prediction of cancer and normal in stomach cancer.

**Figure 2:**
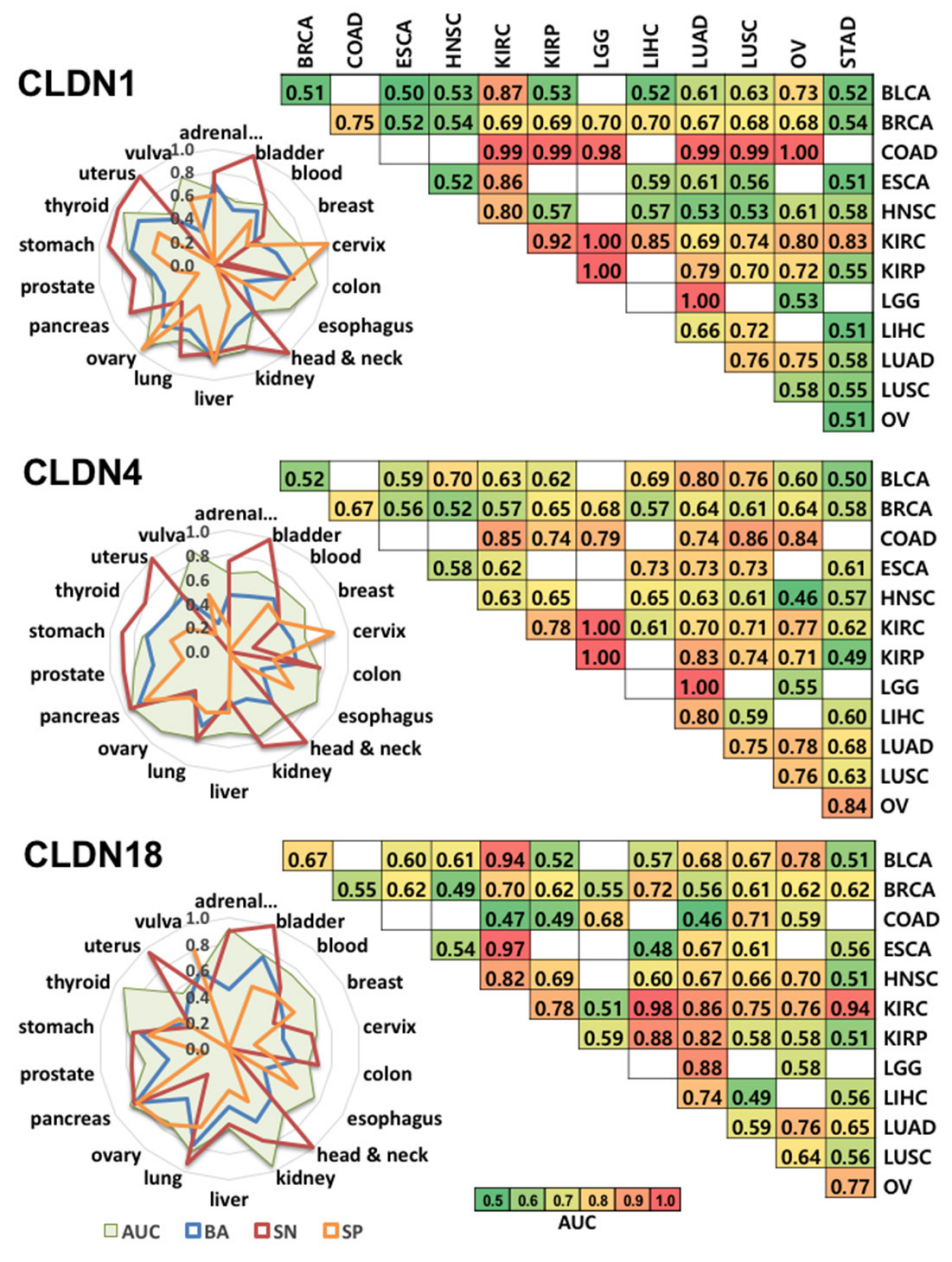
This figure shows CANES evaluation of expression of *CLDN1*, *CLDN4*, and *CLDN18*, as individual biomarkers for discriminating multiple cancer types Left panel is a radial plot representing the predictive value (AUC, BA, SN and SP) of an expressed marker across 18 tumor types within the CANES database. The radial plot shows numeric magnitudes of performance values (0 to 1) from the centers of the circles in 18 radial directions corresponding to the 18 tumor types. In each radial plot, the green area represents AUC, the blue line represents BA, the red line represents SN, and the orange line is SP. For example, the SN for *CLDN4* in bladder cancer is 1.0. The right panel (matrix) represents how well each marker can distinguish between two cancer types. Heatmap color represents the level of AUC. The higher AUC in a heatmap cell indicates that the two pairwise-compared cancer types are better distinguished by a given marker. BLCA, bladder urothelial carcinoma; BRCA, breast invasive carcinoma; COAD, colon adenocarcinoma; ESCA, esophageal carcinoma; HNSC, head and neck squamous cell carcinoma; KIRC, kidney renal clear cell carcinoma; KIRP, kidney renal papillary cell carcinoma; LGG, brain lower grade glioma; LIHC, liver hepatocellular carcinoma; LUAD, lung adenocarcinoma; LUSC, lung squamous cell carcinoma; OV, ovarian serous cystadenocarcinoma. *White box: dataset not yet provided by TCGA.

We next used CANES to predict each of the three *CLDN* genes’ ability to distinguish specific cancers among a panel of 18 tumor types. Figure 2 depicts radial plots (left panel) that represent four performance measures (area under curve; AUC, BA, sensitivity; SN, and specificity; SP) across 18 tumor types per single and/or multiple gene(s). All three claudin genes showed different predictive patterns. AUC plots demonstrated that *CLDN1* and *CLDN18* represent potential predictors of thyroid cancer, and *CLDN4* and *CLDN18*, predictors of pancreatic cancer. Pairwise *CLDN* biomarker AUC comparisons (heatmap, Figure 2, right panel), across 12 tumor types (The Cancer Genome Atlas; TCGA data), showed that *CLDN1* could readily distinguish colon from kidney, brain, lung, and ovary cancers, while both *CLDN1* and *CLDN4* (but not *CLDN18*) could distinguish brain from kidney cancer. While these AUC values would not be sufficiently predictive to discriminate between specific tumor types, it is quite possible that their combination with other highly predictive markers or diagnostic methodologies (e.g., MRI, CT) could reach positive predictive values (PPVs) acceptable for early detection [5, 6].

**Figure 3:**
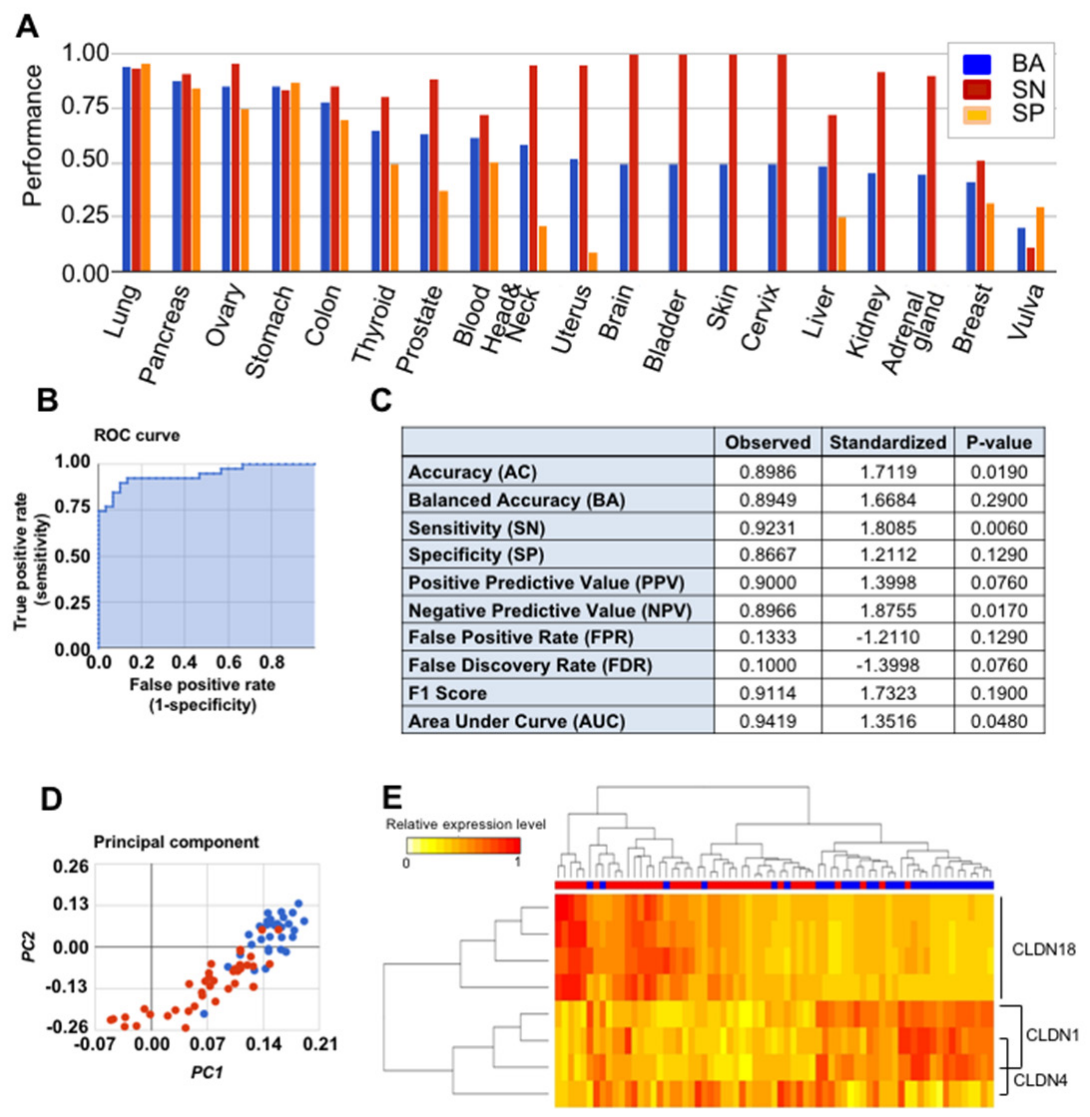
This figure demonstrates CANES evaluation of the performance of a three-member *CLDNs 1/4/18* biomarker panel **(A)** Balanced accuracy (“BA”) (in blue), sensitivity (“SN”) (in red), and specificity (“SP”) (in orange) of the marker panel among 18 different cancer types. **(B)** Receiver operator characteristic (ROC) curve for the predictive accuracy of the panel in a TCGA gastric cancer dataset (GSE13911). AUC is the area under the ROC curve. The higher AUC indicates better performance in terms of both sensitivity and specificity. **(C)** Ten separate evaluation measures of the *CLDN1*/*CLDN4*/*CLDN18* panel for gastric cancer dataset (GSE13911). **(D)** Principal components (PC) analysis (cancerous gastric tissues, red circles; normal gastric tissues, blue circles) showing the separation of cancerous and normal tissues along the first principal component (PC1) and second PC (PC2) in gastric cancer dataset GSE13911. **(E)** Heatmap cluster analysis showing the panel to clearly delineate cancerous vs. normal gastric tissues. The rows are genes or probes, and the columns are cancerous (red horizontal sidebars) and normal tissues (blue horizontal sidebars).

**Figure 4:**
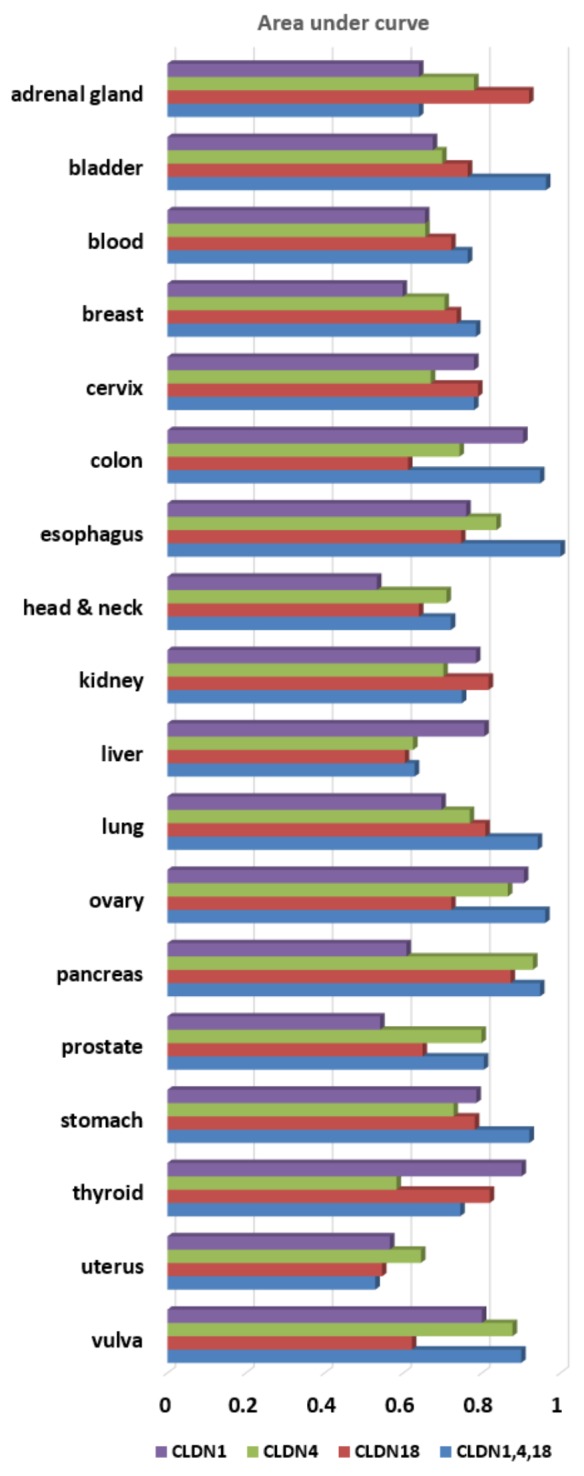
This figure shows a bar plot for diagnostic performances of three single-markers (*CLDN1*, *CLDN4* and *CLDN18*) and a multi-marker (*CLDN1*, *CLDN4* and *CLDN18*) in terms of AUC across 18 tumor types

Based on that (multiple biomarker) hypothesis, we evaluated the three claudin family genes as a multi-marker. Figure 3A shows the multi-marker performances of *CLDN1*, *CLDN4*, and *CLDN18* expression in distinguishing 18 tumor types. Figure 3B-3E show multi-marker performances in GC. Figure 3C shows higher values for the three-gene set, as compared to single marker performance in GC (Table 1). When *CLDN1*, *CLDN4*, and *CLDN18* were analyzed, as single markers, across 18 cancer tissue types, the AUC values were 0.756 (p=1.4×10^-4^), 0.647 (p=0.156), and 0.792 (p=2.5×10^-4^), respectively. When *CLDN1*, *CLDN4*, and *CLDN18* were analyzed throughout the 18 cancer types as a multi-marker set, the AUC value was 0.850 (p=3.3×10^-4^) (Table 1 and Figure 4). Thus, these findings support the many assertions that multiple biomarker sets hold greater sensitivity/specificity, compared to single markers, for disease detection [5, 6], in general or at-risk populations. To address the problem that a randomly chosen marker set with a large number of probes often tends to show good performance, CANES provides empirical p-values.

**Figure 5:**
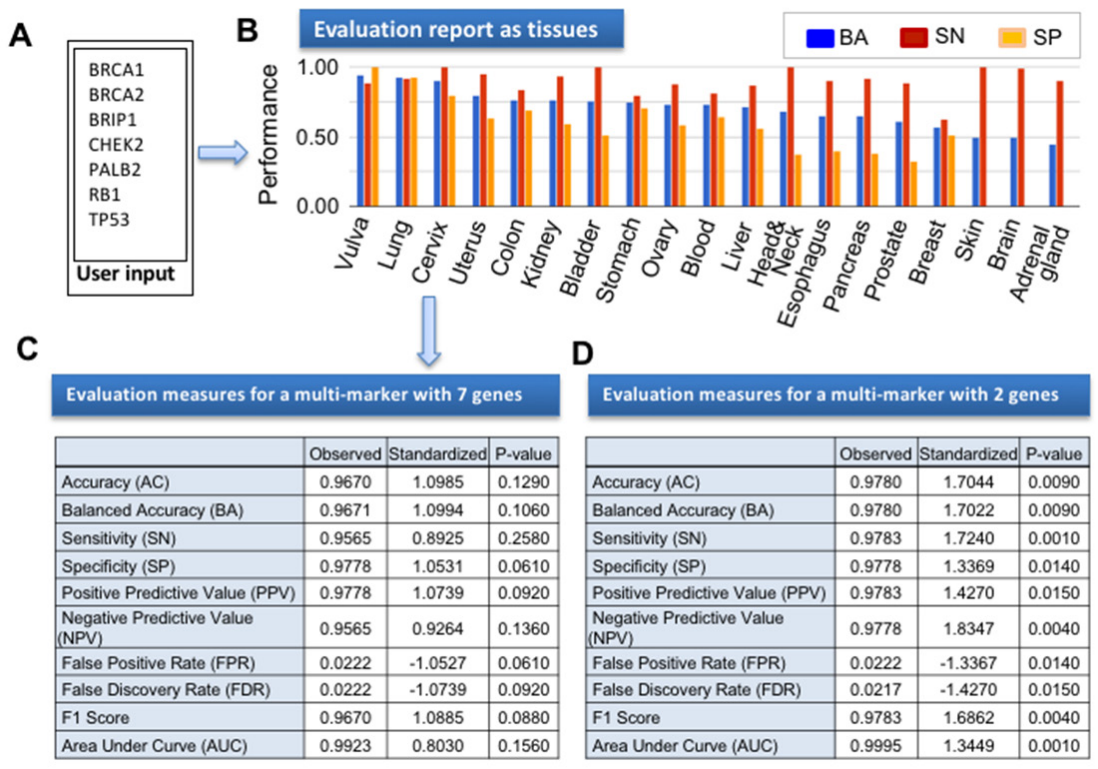
The figure here shows an example CANES output report following upload of breast cancer biomarker set **(A)** In this instance, seven well-known breast cancer biomarkers (*BRCA1*, *BRCA2*, *BRIP1*, *CHEK2*, *PALB2*, *RB1*, and *TP53*) were evaluated for numerous performance measures in lung cancer. **(B)** Bar graph of three evaluation measures (BA, SN, and SP) of the 7-member panel across 18 organs. **(C)** The numerical evaluation measures of the 7-member panel in a lung cancer dataset (GSE18842). **(D)** The evaluation measures for a multi-marker with *BRIP1* and *RB1* in GSE18842.

In addition to evaluating biomarker performance for specific tumors, another key feature of CANES is its assessment of the predictive accuracy of multiple marker panels, among multiple cancer types. Consequently, we evaluated a panel of well-known breast cancer markers *BRCA1*, *BRCA2*, *BRIP1*, *CHEK2*, *PALB2*, *RB1* [21], and *TP53* [22], for predictive accuracy in lung cancer. Figure 5 shows the CANES evaluation report for those seven grouped breast cancer biomarkers, as classified by support vector machine and leave-one-out cross-validation, using lung cancer datasets from 46 cancer and 45 normal tissues [23] as the testing dataset. Figure 5 shows a representative CANES performance report on the test dataset. The seven multi-markers (Figure 5A) were evaluated in multiple cancer types (Figure 5B), and showed higher AUC, BA, SN, and SP values for lung cancer (Figure 5C). However, this multi-marker set was not statistically significant (p=0.129 for BA and p=0.156 for AUC) in lung cancer. To find significant multi-marker sets, all possible subgroups of the seven genes were evaluated using the same lung cancer dataset. Finally, 10 subgroups had significant BAs and AUCs, among which a multi-marker set with *BRIP1-RB1* showed the best performance, with a BA=0.9780 (p=0.009) and AUC=0.9995 (p=0.001) (Figure 5D). After adjusting for multiple testing, using the Westfall and Young multiple correction method [24], the adjusted p-values were 0.055 and 0.024 for BA and AUC, respectively. Based on this performance evaluation, these two biomarkers could potentially be applied to lung cancerdiagnostic evaluations, similar to a previous report that in addition to breast cancer, the oncogene *ERBB2-HER2* is amplified in subgroups of glioblastomas and stomach, uterine, bladder, and lung cancers (thus suggesting possible repurposing of the anti-HER2 antibody trastuzumab for these cancers) [10]. This result demonstrates that transcriptomic analysis of molecular patterns across cancer types allows the etiologic and therapeutic knowledge of one cancer type to be applied to another, suggesting that therapy guidance/response markers for one tumor may also be appropriate for others. Therefore, CANES provides powerful prediction to evaluate biomarkers across cancer types.

In addition to the above, we previously reported five genes, *ENAH, RAD51, CHEK2, ATF4*, and *ICOSLG*, as possible drug response biomarkers in breast cancer [25]. Setting these genes as a reference set in breast cancer, we compared biomarker suggestion results for the five candidates by using each tool (Table 2). One widely used commercial tool, Ingenuity Pathway Analysis (IPA), does not report a numerical representation for performance evaluation, including AUC, except either detection or no detection for single genes [26]. Also, IPA cannot perform multi-gene biomarker performance evaluation, and relies on its own database [27]. Similarly, Oncomine merely reports limited quantified information, such as the number of significant differential analyses (driven by Student t-tests for two classes) relating to each candidate biomarker [28]. However, Oncomine does not describe evaluation quantification for multi-gene biomarker performance, and is restricted to microarray analysis [29]. Unlike these two tools, CANES reports diverse performance evaluations (only AUC shown due to limited space in Table 2) for multi-gene biomarkers, as well as for each candidate. For example, considering that AUC > 0.75 supports good biomarker feasibility, *ENAH* and *RAD51* could be repurposed for breast cancer diagnosis usage. Thus, CANES can introduce biomarker candidates from published literature for diagnosis of other cancer types, based on evidence-based measurements.

**Table 2:** Comparison of 5 biomarkers identified as Herceptin non-responsive biomarker between CANES vs. IPA-biomarker and Oncomine V4.5

	CANES			IPA-biomarker			Oncomine v4.5		
	Single biomarker evaluation (AUC^1^)	Multi-gene biomarker evaluation (AUC^1^)	Quantification of single/multi-gene biomarker	Single biomarker evaluation	Multi-gene biomarker evaluation^2^	Quantification of single/multi-gene biomarker	Single gene evaluation: detection rate (A/B^3^)	Multi-gene biomarker evaluation^2^	Quantification of single/multi-gene biomarker
*ENAH*	0.826	0.854	Yes (11 measurements)	Yes	-	No	11.32 % (6/53)	-	Only single biomarker with limited quantification
*RAD51*	0.777			Yes			9.09 % (4/44)		
*ATF4*	0.688	0.78		No	-		1.96 % (1/51)	-	
*CHEK2*	0.681			No			2.33 % (1/43)		
*ICOSLG*	0.653			No			0 %		

Previously, we proposed the five genes *ENAH, RAD51, CHEK2, ATF4*, and *ICOSLG* as Herceptin non-responsive biomarker candidates in breast cancer. Setting the genes as a reference set in breast cancer, we inspected the agreement between the five genes and each tool’s results. IPA-biomarker does not report a numerical representation for evaluation except either detection or no detection. Oncomine reports the number of significant differential analyses relating to each candidate, without performance evaluation information. For comparison of multi-gene biomarker evaluation, we used the two multi-gene biomarker sets (one for *ENAH* and *RAD51*; the other for *ATF4, CHEK2*, and *ICOSLG*).

^1^AUC: average of area under curve.

^2^IPA and Oncomine do not support a multiple-gene biomarker evaluation (NA: non applicable).

^3^In breast cancer, ”A” represents the number of total analyses relating to a given gene, and “B” the number of significant analyses relating to the gene. For example, in *ENAH*, Oncomine reports the 53 analyses relating to *ENAH* in breast cancer. Out of them, six analyses reports the significant expression difference between breast cancer and normal groups.

### Dynamic usage of big-data-based for predicting mutli-biomarker system for oncology therapeutic development

The utility of CANES extends beyond the above illustrations. For example, CANES supports the feasibility (e.g., high sensitivity and specificity) of using specific panels of biomarkers for widespread population screening for distinct cancer types (or at minimum, to individuals already at increased risk for such cancer types), representing the achievement of a previously extremely difficult endeavour [30, 31]. We concede that biomarker discovery using CANES represents merely one step in a long and arduous process [2, 32], according to the recently adopted REporting recommendations for tumor MARKers (REMARK) guidelines [2]. However, should the newly discovered, tumor-specific gene expression biomarkers prove present in body fluids, improved preclinical accuracy could potentially enhance the eventual translation of such diagnostics [3] toward the long-desired goal of simple blood or urine tests for cancer detection in high-risk populations [33, 34]. Moreover, from a research perspective, identifying a strong association of a specific gene(s) with a particular tumor could facilitate understanding of the mechanism-of-action(s) of that specific biomarker(s), and the identification of other druggable targets/pathways involved in the progression of that distinct tumor.

Thus, CANES represents a novel and publically available tool for enhancing the characterization/discovery of single/multi-biomarker sets for specific cancer types. This tool will also provide analysis to implement within translational research, improving the characterization of specific cancer types, identifying cancer progression pathways, and improving evidence-based biomarker therapeutic development.

Unlike other diseases, the development of clinical cancer biomarkers has been fraught with difficulties [31, 35]. Despite several thousands of publications, the actual number of clinically approved biomarkers remains less than 100 [3, 30]. For general population screening, the prostate-specific antigen (PSA) remains the only approved serum biomarker, and guidelines even for its use have spurred controversy (e.g. men over age 40 vs. 50, etc., non-family prostate cancer history, etc.), due to its high false positive rate, and subsequently, unnecessary, invasive procedures [36]. Similarly, while tumor whole genome and transcriptome sequencing have ushered in the advent of “personalized” therapies, individualized for specific patients, the cost/benefit of these massive analyses remains debatable, and these approaches may be confounded by uncontrolled false discovery rates and the genomic instability and heterogeneity found in most tumors [37]. Likewise, while the clinical utility of tumor-specific prognostic gene expression “signatures” has gained greater acceptance [5], many have not yet proved unreliable [38]. Even for well-known prognostic biomarkers, such as the Cancer Embryonic Antigen (CEA, colon cancer), CA-125 (ovarian cancer), and CA-19-9 (pancreatic cancer), their precise role(s) in the progression of those diseases remains largely unknown [30]. Moreover, the poor “bench-to-bedside” progression of preclinically discovered biomarkers has been attributed to a number of factors, including biased or low-rigor statistical assessment, irreproducibility, and an overall decreased quality of preclinical studies [39]. Despite a number of ambitious attempts to remedy these shortcomings [2, 32, 40], this overall trend has largely persisted [30, 31, 35].

## DISCUSSION

One possible solution to increasing biomarker success rates is through the use of bioinformatics and improved statistical evaluation, using publically accessible databanks, thus increasing sample sizes and removing various confounding variables [3, 41, 42]. In this study, we undertook such an approach by designing a single/multi-biomarker evaluation tool, CANES, a simple and user-friendly web-based application. CANES evaluates multiple markers, using the above-described data repositories, to harness the power of “Big Data” for researchers to develop new models of translational research, for diagnostic and prognostic applications, and “targeted” therapies. By incorporating clinical data from those databases, matched to specific patient transcriptomes/genomes, CANES can evaluate the performance of multiple biomarkers for a number of clinical parameters, e.g., diagnosis, therapy response, survival, etc. (in contrast to other widely used analysis tools), thus increasing the robustness of assessment (for improved screening) and improving the probability of eventual clinical translation [30, 31]. While CANES continues to use all publicly available microarray datasets, it can also incorporate next generation sequencing technology (e.g., RNA-seq) datasets, for specific cancers, that are now increasingly available from the TCGA [10] and other databases.

Currently, CANES provides classification evaluation of numerous organ-based cancer types, including liver, lung, and pancreatic cancers, and many others. For each cancer subtype, even though there is considerable publically available expression data, with subtype information, CANES cannot yet support subtype-based classification evaluation, due to subtype’s term diversity and lack of standardization of subtype terms. However, we have now designed a plan to update our system for subtype-based classification evaluation.

In summary, CANES is a powerful tool that will enable preclinical researchers to assist bench-side researchers in exploring available data for the disease of interest, as well as cater to the needs of bedside practitioners, to develop and implement cancer-specific biomarker therapies.

## MATERIALS AND METHODS

### Web-based *CAN*cer-specific single/multi-biomarker *E*valuation *S*ystem (CANES)

**Figure 6:**
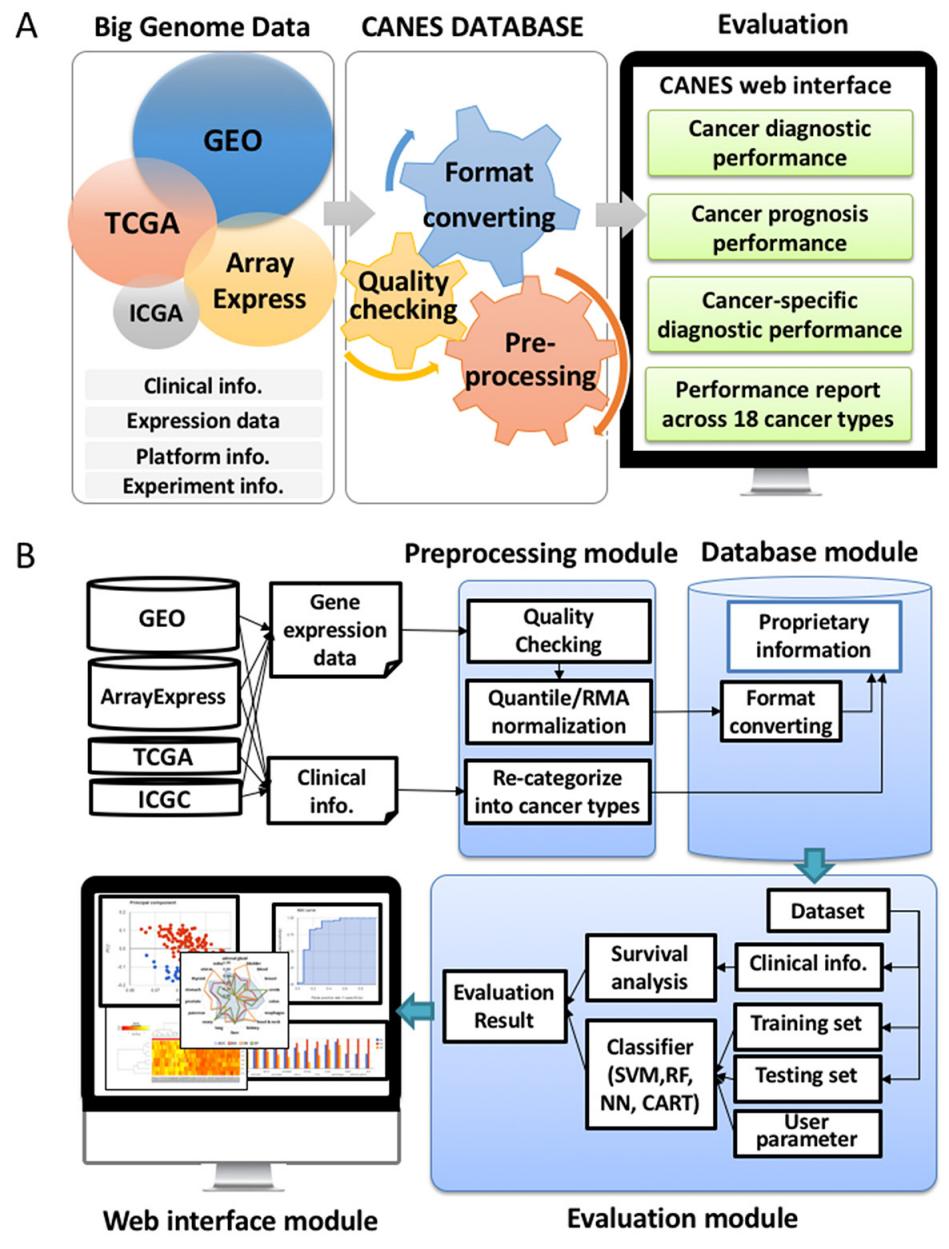
Key features of CANES **(A)** The figure depicts how the web-based CANES tool provides a highly stringent evaluation system to compare user input marker candidates to datasets retrieved from four “Big Data” depository (TCGA, GEO, ArrayExpress, ICGC). For reproducibility of evaluation, CANES not only constructs its own preprocessed expression datasets but also provides various evaluation analyses (cancer diagnosis and prognosis). **(B)** For user inquires, CANES consists of four modules (1) a preprocessing module (a step for data normalization, as well as for clinical information summary); (2) a database module (for storing pre-calculated data); (3) an evaluation module (a step for evaluating users’ input genes based on survival significance and classification performance); and (4) a web-interface module (for user-friendly visualization).

CANES postulates that biomarker candidates are well reproduced in multiple, independent datasets, regardless of different technology platforms (e.g., RT-PCR, microarrays, RNA-Seq, etc.). CANES processes individual datasets in a preprocessing step (without merging all the datasets into a single pool). The evaluation phase then inspects whether or not the biomarker candidates are reproduced across multiple samples. CANES collects RNA molecular profiles from public databases and assigns them into distinct tumor types using their annotations, following a rigorous quality control process. CANES then provides evaluation results for user-specified, multiple markers, across various cancer types or studies. As shown in Figure 6, CANES has four modules: a preprocessing module, a database module, an evaluation module, and a web-interface module. The preprocessing module normalizes individual datasets separately for storage in the CANES internal database, which is then used as an expression resource for evaluation. For the selected biomarker candidate (single and/or multi-gene), the evaluation module provides numerous measures for assessing prediction performance.

**Figure 7:**
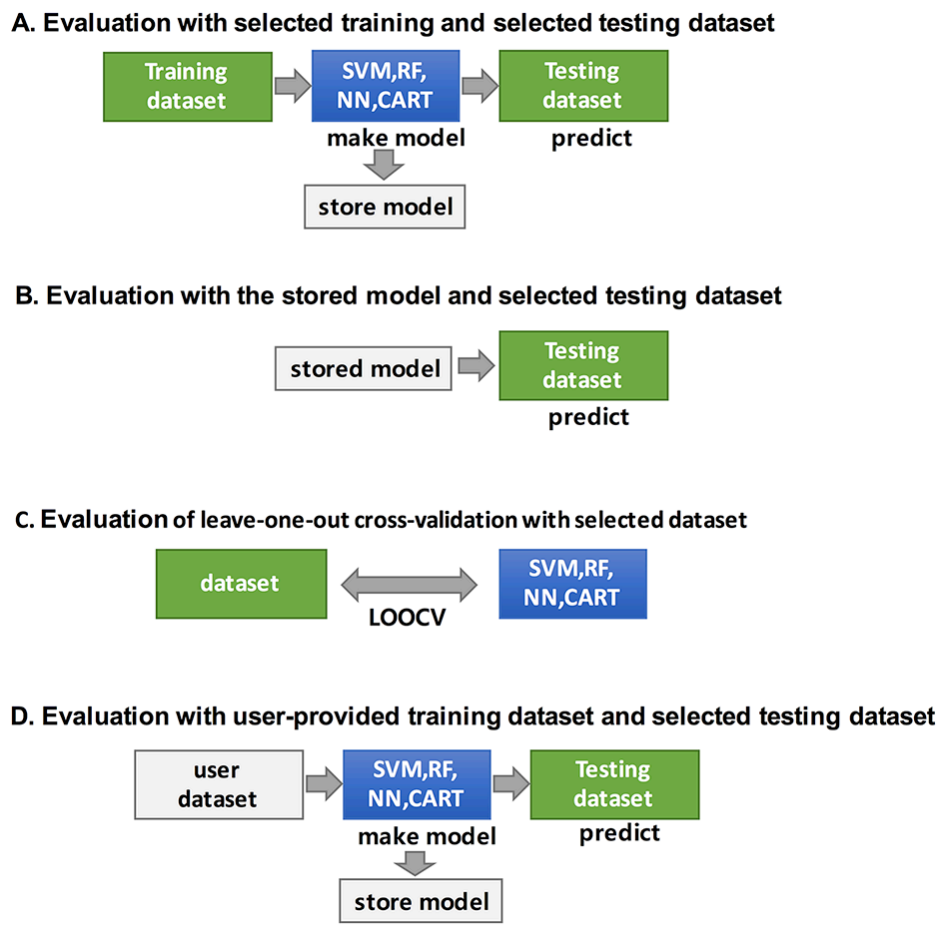
The figure shows how CANES performs the four classifications of the biomarker candidate evaluations SVM, support vector machine; RF, random forest; NN, neural network; CART, a classification tree. Classifications are used to evaluate the performances power.

Figure 6A shows a schematic of CANES’ overall procedure. The CANES database draws information, from 18 distinct tumor type datasets, from “big genome” data depositories, including the Gene Expression Omnibus (GEO) [44], TCGA [10], the International Cancer Genome Consortium (ICGC) [45, 46], and ArrayExpress, a functional genome database administered by the European Bioinformatics Institute [43]. After steps involving quality checking, format conversion (to match the user’s biomarker search entry(ies)), and preprocessing, CANEs evaluates the performance of single/multi-biomarker candidates, based on four established classification methods (Figure 7A-7D).

CANES evaluation can also be based on a stored model, trained by various classifiers, to assess the predictive value of a test dataset (Figure 7B). Alternatively, the user can provide his/her own training dataset to train a model using the same four classifiers (Figure 7D). For classification, CANES employs a consensus of four different approaches, support vector machine (SVM), random forest (RF), neural networks (NN), and classification and regression trees (CART). For validation of user-provided datasets, CANES uses leave-one-out cross-validation (LOOCV) (Figure 7C).

Figure 6B depicts a flowchart to further illustrate the CANES pipeline. The user uploads a marker candidate set through a web-interface module, entering the candidate set that is evaluated against clinical, preprocessed, and normalized gene expression data, that is then recategorized from the four above-mentioned public data repositories (Figure 6B). Based on user-defined cut-off levels for high vs. low gene expression, CANES evaluates the biomarker(s) for the following measures: AUC (an accuracy measurement based on the true positive rate plotted as a function of the false negative rate) [42], accuracy (AC), BA (defined as the arithmetic mean of SN and SP that corrects for imbalanced performance by the classifiers) [47], SN, SP, positive predictive value (PPV), negative predictive value (NPV), false positive rate (FPR), false discovery rate (FDR), and F1 score (a test accuracy measurement that considers both precision and recall) (Supplementary Table 1) [47].

Table 3 shows the notable advantages of CANES over other widely used biomarker database analyses, including Oncomine [48], IPA-Biomarker (www.qiagen.com/ingenuity), and cBioPortal [49], in terms of biomarker evaluation functionality. These include: 1) survival analysis, including Kaplan-Meier analysis and Cox proportional hazard regression; 2) evaluation of mature miRNAs as markers, as well as genes, for user-defined diagnostic or prognostic purposes; and 3) provision of a pan-cancer summary view for evaluating each single marker (Table 3).

**Table 3:** Comparison of databases relating to biomarker evaluation

Functions		CANES	ONCOMINE	IPA-biomarker	cBioPortal
**Performance evaluation for single marker**	**Pan-cancer summary view**	Yes (Reporting all the BAs and AUCs across pan-cancer)	Yes (reporting the number of over/under expressed genes in pan-cancer)	No	Yes (only proportion of alterations only applied across pan-cancer)
	**Survival analysis**	Yes	No	No	Yes
	**Utility for miRNA**	Yes	No	Yes	No
**Performance evaluation for multiple markers**	**Pan-cancer summary view**	Yes	No	No	No
	**Survival analysis**	Yes	No	No	No
	**Utility for miRNA**	Yes	No	No	No
**Accessibility**		Free	Free for non-profit organisations but allow restricted use	Commercial	Free
**User dataset is mandatory?**		No	No	Yes	No

### Preprocessing module

The current version of CANES uses microarray data obtained from two public repositories and two cancer consortia, GEO [44], ArrayExpress [43], TCGA [10], and ICGC [46]. All expression datasets can be collected using the R package GEOquery [50]. In the CANES preprocessing module, expression datasets from these public repositories are parsed and normalized by quantiles robust multi-array average (RMA) [51]. For RNA sequencing data, counts are normalized to expression values. All datasets with missing rates > 5% are excluded, and the remaining datasets with missing values are imputed by the ‘impute’ package [52] of Bioconductor. To detect outliers caused by instrument error or sample contamination, mislabeling, or misprocessing, we use within-group and between-group correlations [53]. Since all detected outlier samples are marked, users can exclude them from their analyses, using specified options. Moreover, available clinical information and sample annotations are parsed into the CANES database. To define the exact cancer type or subtype, we confirm or recategorize diagnoses, prognoses, and drug responses as designs of the dataset. All processed expression data are converted into customized, (Figure 6B, middle) indexed binary files for fast retrieval as big genomic expression data. The preprocessing module is implemented using Python and R.

### Database module

The database module contains preprocessed expression datasets and their corresponding annotation data. Currently, the preprocessed datasets consist of gene expression data and annotation data for 94,147 samples (Supplementary Table 2). Gene expression data are obtained from these samples with broadly used gene expression microarray platforms and RNA sequencing platforms, and processed as described. The database module is implemented using MySQL and Python. All expression data is saved as customized indexed binary files.

### Evaluation module

The evaluation module of CANES is implemented using classification methods such as SVM, RF, NN, and CART. For user-selected multiple markers, this module provides the evaluation result with the evaluation measures across 18 cancer-types, based on ten evaluation measures, including AUC, AC, BA, SN, SP, PPV, NPV, FPR, FDR, and F1 score [47] (Supplementary Table 1). In addition, the evaluation module provides standardized evaluation measures and empirical p-values to address the problem of randomly chosen marker sets, with a large number of probes, tending to show good performance (for further description in “Summary evaluation measurements and their p-value calculations in CANES” in the Materials and Methods section). To measure the contribution of a single marker to the performance of multi-markers, CANES provides an influence measure, which is the difference between the evaluation measure for all markers and that for all markers excluding the single marker. All these manipulations can be conducted by four different evaluation schemes, as follows (Figure 6B).

*i) Evaluation of multiple markers with selected training and testing datasets*. CANES can conduct prediction analysis using specific cancer types or studies. Users can generate and store the prediction model for their own multi-marker lists using the selected dataset and classification method. Graphical and interactive result layouts are provided and can be saved.

*ii) Evaluation using a stored prediction model on a selected testing dataset*. CANES stores the evaluation result, which can then be used on a different testing dataset. For example, users can store the prediction model with breast cancer markers and breast cancer datasets, and then evaluate this stored model against a liver cancer dataset.

*iii) Evaluation of LOOCV with the selected dataset*. To prevent overfitting by any specific training dataset, CANES can evaluate multiple markers using LOOCV. In this evaluation module, CANES can also support the evaluation of individual markers, in a multi-marker set, by measuring the contribution level of the performance of multi-markers.

*iv) Evaluation with the user-provided training dataset and the selected testing dataset*. CANES allows evaluation of a prediction model generated by a user-provided dataset. The user dataset is uploaded via a web-interface module, is preprocessed and normalized, and is then used as a training dataset employingdifferent classification methods. The prediction models trained with the user’s own dataset are tested with independent datasets from public repositories.

### Web-interface module

The web-interface of CANES consists of the input layout and the result explorer. The input layout is the interface that transfers user-selected multiple markers and queried parameters to the evaluation module. In the input layout, a user can input a set of official gene symbols, miRNAs, or probe IDs, and select either a preprocessed public dataset, or a user-uploaded private dataset, as the training dataset. The result explorer provides tables and graphical visualization of the evaluation results (Figure 6B, bottom left). The CANES web-interface module is implemented using PHP, within a JQuery and CodeIgniter framework.

CANES is freely accessible from the CANES website http://bibs.snu.ac.kr/software/canes. Moreover, the design and implementation of CANES facilitates easy incorporation of additional query functions and applications, as well as other datasets, irrespective of cancer type, in the form of pre-processed datasets. All evaluation results are presented in a table and/or graphical visualization, and can be downloaded as high-quality PDF images and CSV-based text-format spreadsheets.

### Summary evaluation measurements, and their p-value calculations, in CANES

We defined various evaluation measurements, in consistency with widely accepted formulas, as follows: accuracy(AC) is (TP+TN)/(TP+TN+FP+FN) where TP is the true positive value, TN the true negative, FP the false positive, and FN the false negative value. Sensitivity (SN) is defined as TP/(TP+FN), and specificity (SP) by TN/(FP+TN). In addition, balanced accuracy (BA) is defined as (SN+SP)/2, while positive predictive value (PPV) is defined as TP/(TP+FP), negative predictive value (NPV) as TN/(FN+TN), false discovery rate (FDR) as 1-PPV, and F1 as 2TP/(2TP+FP+FN). Area under the curve (AUC) is the area under the receiver operating characteristic (ROC) curve, which is the line representing the true positive rate (TPR or sensitivity) and false positive rate (FPR or 1-specificity) of any distinct diagnostic test. AUC can also be used as an index of the test’s performance (Supplementary Table 1).

Based on these evaluation measures, CANES provides standardized evaluation measures, and empirical p-values, as follows:

i) CANES calculates the observed evaluation measure (*t*_*i,o*_) for the user-defined marker set with *m*_*i*_ probes for the *i*_*th*_ dataset.

ii) CANES uses a preconstructed empirical null distribution of the observed evaluation measure (*t*_*i,o*_). In the *i*_*th*_ dataset, *n* probe sets are generated with randomly selected *m*_*i*_ probes. The empirical null distribution is then constructed using the evaluation measure (*t*_*i,r*_) for the *n* probe sets.

iii) Using the empirical null distribution, the average ($\bar{t_{i}}$) and the standard deviation (*s*_*i*_) can be calculated as follows:$s_{i}$

$$\bar{t_{i.}}=\frac{\sum_{r=1}^{n}t_{i,r}}{n}\;and\;s_{i}=\sqrt{\frac{\sum_{r=1}^{n}{\left(t_{i,r}-\bar{t_{i.}}\right)}^{2}}{n-1}}$$

The standardized evaluation measure (*Z*_*i*_), and its p-value, are defined as follows:

$$z_{i}=\frac{t_{i,o}-\bar{t_{i.}}}{S_{i}}and\;p_{i}=\frac{\sum_{r=1}^{n}I\left(t_{i,r}\geq t_{i,o}\right)}{n}$$

Note that $\bar{t_{i}}$ and *s*_*i*_ need to be computed for *m*_*i*_ probes in the *i*_*th*_ dataset.

There is an advantage in using *z*_*i*_ over *t*_*i,o*_. We found out that the values of *t*_*i,o*_ tend to increase as the number of probes increases. Thus, *t*_*i,o*_ needs to be standardized to be sufficiently robust to the number of probes. *z*_*i*_ is a standardized version of *t*_*i,o*_, and is a more appropriate evaluation measure than *t*_*i,o*_. Through *z*_*i*_, a direct comparison of the diagnostic performance between models with different numbers of probes becomes possible. The empirical p-value is the relative frequency that the randomly selected marker sets have better performances than the user-defined marker set. Note that these p-values are the same for *z*_*i*_ and *t*_*i,o*_.

To summarize performance measures from *k* datasets into a single measure, CANES provides the summarized p-value by combining p-values as follows:

i) minP method : $P_{A}=\min\left\{p_{1},p_{2},\ldots ,p_{k}\right\}$

ii) Fisher’s method : $P_{A}=1-\chi_{2k}^{2}\left(-2\sum_{i=1}^{k}\ln\left(p_{i}\right)\right)$

iii) Stouffer’s method :$P_{A}=1-\Phi \left(\frac{\sum_{i=1}^{k}Z_{i}}{\sqrt{k}}\right),$ when *Z* = Φ^−1^ (1 − *p*_*i*_)

iv) Weighted Stouffer’s method : $P_{A}=1-\Phi {\left(\frac{\sum_{i=1}^{k}w_{i}Z_{i}}{\sqrt{\sum_{i=1}^{k}w_{i}^{2}}}\right)}_{,\;when\;}w_{i}=\sqrt{4/\left(\frac{1}{n_{i}^{case}}+\frac{1}{n_{i}^{control}}\right)}$

### System implementation of CANES

The job scheduling scheme of CANES is based on a first-come, first-serve process. To support intensive queries from public users, the CANES system consists of one web-server and 10 Xeon® (manufactured by Intel) calculation servers. Once a job is submitted by the user, the job is executed in the background, on calculation servers. Therefore, users don’t need to keep the submission webpage open on their browser until the job is finished. After the user’s job is done, CANES sends a notice email with a direct link to the results page. To prevent waste of computing resource by redundant model fitting, CANES can keep the previous search results in the cache space and provide the stored results, without re-evaluation.

## SUPPLEMENTARY MATERIALS TABLES







## Abbreviations

AUC: area under curve
BA: balanced accuracy
CANES: CANcer-specific single/multi-biomarker Evaluation System
CART: classification and regression trees
CEA: Cancer Embryonic Antigen
EGFR: epidermal growth factor receptor
FDR: false discovery rate
FN: false negative value
FP: false positive value
FPR: false positive rate
GC: gastric cancer
GEO: Gene Expression Omnibus
ICGC: the International Cancer Genome Consortium
LOOCV: leave-one-out cross-validation
NN: neural networks
NPV: negative predictive value
PPV: positive predictive value
PSA: prostate-specific antigen
REMARK: REporting recommendations for tumor MARKers
RF: random forest
RMA: robust multi-array average
ROC: receiver operating characteristic
SN: sensitivity
SP: specificity
STRING: Search Tool for the Retrieval of Interacting Genes/Proteins
SVM: support vector machine
TCGA: The Cancer Genome Atlas
TN: true negative value
TP: true positive value
