# Supplementary material for: CANcer-specific Evaluation System (CANES): a high-accuracy platform, for preclinical single/multi-biomarker discovery
Source: Oncotarget. 2017 Jul 15;8(41):69808–22. doi: 10.18632/oncotarget.19270 (PMC5642518; doi:10.18632/oncotarget.19270)
Supplement: Supplementary file 1 [file oncotarget-08-69808-s001.pdf]

CANcer-specific Evaluation System (CANES): a high-accuracy platform, for preclinical single/multi-biomarker discovery

SUPPLEMENTARY MATERIALS

Supplementary Table 1: Evaluation measures in CANES

See Supplementary File 1

Supplementary Table 2: Number of samples in CANES

See Supplementary File 2
