# Supplementary material for: CANcer-specific Evaluation System (CANES): a high-accuracy platform, for preclinical single/multi-biomarker discovery
Source: Oncotarget. 2017 Jul 15;8(41):69808–22. doi: 10.18632/oncotarget.19270 (PMC5642518; doi:10.18632/oncotarget.19270)
Supplement: Supplementary file 2 [file oncotarget-08-69808-s002.docx]

**Supplementary Table 1: Evaluation measures in CANES**

| Measure | Description |
| --- | --- |
| Area under curve (AUC) | AUC is the area under the receiver operating characteristic (ROC) curve, which is the line with true positive rate (TPR or sensitivity) and false positive rate (FPR or 1-specificity) of diagnostic test. AUC can also be used as a summary index of the test’s performance. |
| Accuracy (AC) | $AC=\frac{TP+TN}{TP+TN+FP+FN}$ |
| Balanced accuracy (BA) | Balanced accuracy is the arithmetic mean of sensitivity and specificity. It avoids inflated performance estimates on imbalanced datasets.  $BA=\frac{1}{2}\left( SN+SP \right)=\frac{TP}{2(TP+FN)}+\frac{TN}{2(FP+TN)}$ |
| Sensitivity (SN) | $SN=\frac{TP}{TP+FN}$ |
| Specificity (SP) | $SP=\frac{TN}{FP+TN}$ |
| Positive predictive value (PPV) | $PPV=\frac{TP}{TP+FP}$ |
| Negative predictive value (NPV) | $NPV=\frac{TN}{FN+TN}$ |
| False discovery rate (FDR) | $FDR=\frac{FP}{TP+FP}=1-PPV$ |
| F1 score (F1) | F1 score is the harmonic mean of precision and sensitivity.  $F1=\frac{2TP}{2TP+FP+FN}$ |

TP: true positive, FP: false positive, TN: true negative, FN: false negative
