# Supplementary material for: CANcer-specific Evaluation System (CANES): a high-accuracy platform, for preclinical single/multi-biomarker discovery
Source: Oncotarget. 2017 Jul 15;8(41):69808–22. doi: 10.18632/oncotarget.19270 (PMC5642518; doi:10.18632/oncotarget.19270)
Supplement: Supplementary file 3 [file oncotarget-08-69808-s003.docx]

**Supplementary Table 2: Number of samples in CANES**

| **no** | **tissue** | **no. of cancer** | **no. of normal** | **no. of cell line** | **total** |
| --- | --- | --- | --- | --- | --- |
| 1 | abdomen | 51 | 0 | 0 | 51 |
| 2 | adipose | 4 | 234 | 0 | 238 |
| 3 | adrenal gland | 55 | 20 | 0 | 75 |
| 4 | bladder | 154 | 55 | 119 | 328 |
| 5 | blood | 26806 | 3132 | 1747 | 31685 |
| 6 | bone | 0 | 0 | 48 | 48 |
| 7 | brain | 3109 | 2645 | 661 | 6415 |
| 8 | breast | 9324 | 1057 | 2946 | 13327 |
| 9 | cervix | 447 | 48 | 94 | 589 |
| 10 | colon | 7282 | 1137 | 566 | 8985 |
| 11 | connective tissue | 0 | 0 | 154 | 154 |
| 12 | endometrium | 285 | 297 | 0 | 582 |
| 13 | esophagus | 190 | 36 | 48 | 274 |
| 14 | eye | 249 | 0 | 21 | 270 |
| 15 | head neck | 800 | 55 | 0 | 855 |
| 16 | kidney | 2823 | 515 | 115 | 3453 |
| 17 | liver | 768 | 198 | 231 | 1197 |
| 18 | lung | 2166 | 1315 | 3418 | 6899 |
| 19 | lymphoma | 0 | 0 | 632 | 632 |
| 20 | muscle | 0 | 859 | 40 | 899 |
| 21 | myeloma | 0 | 0 | 28 | 28 |
| 22 | ovary | 3572 | 202 | 95 | 3869 |
| 23 | pancreas | 689 | 249 | 198 | 1136 |
| 24 | pharynx | 0 | 0 | 24 | 24 |
| 25 | placenta | 0 | 0 | 46 | 46 |
| 26 | prostate | 812 | 150 | 78 | 1040 |
| 27 | rectum | 0 | 0 | 53 | 53 |
| 28 | sarcoma | 4059 | 0 | 240 | 4299 |
| 29 | skin | 1156 | 550 | 711 | 2417 |
| 30 | small intestine | 51 | 24 | 0 | 75 |
| 31 | spleen | 0 | 24 | 0 | 24 |
| 32 | stomach | 1485 | 226 | 317 | 2028 |
| 33 | synovial membrane | 0 | 0 | 12 | 12 |
| 34 | testis | 16 | 36 | 0 | 52 |
| 35 | thyroid | 246 | 99 | 348 | 693 |
| 36 | tongue | 0 | 44 | 0 | 44 |
| 37 | uterus | 614 | 48 | 483 | 1145 |
| 38 | vagina | 12 | 20 | 0 | 32 |
| 39 | vulva | 83 | 55 | 36 | 174 |
|  | total | 67308 | 13330 | 13509 | 94147 |
